# Comparative Analysis of Cold Versus Thermal Dissection in Nerve-Sparing Robot-Assisted Radical Prostatectomy

**DOI:** 10.3390/cancers17111831

**Published:** 2025-05-30

**Authors:** Andrea Fuschi, Manfredi Bruno Sequi, Yazan Al Salhi, Paolo Pietro Suraci, Fabio Maria Valenzi, Onofrio Antonio Rera, Alice Antonioni, Damiano Graziani, Giorgio Martino, Giuseppe Candita, Filippo Gianfrancesco, Paolo Benanti, Luca Erra, Giovanni Di Gregorio, Riccardo Lombardo, Anastasios D. Asimakopoulos, Cosimo De Nunzio, Felice Crocetto, Matteo Pacini, Eleonora Sollazzi, Alessandro Zucchi, Antonio Carbone, Antonio Luigi Pastore

**Affiliations:** 1 Urology Unit, Department of Medico-Surgical Sciences and Biotechnologies, Faculty of Pharmacy and Medicine, Sapienza University of Rome, 04100 Latina, Italy; 2 Department of Urology, Faculty of Medicine and Psychiatry, Sant’Andrea Hospital, 00189 Rome, Italy; 3 Unit of Urology, Department of Surgical Sciences, Tor Vergata University Hospital, 00133 Rome, Italy; 4 Department of Neurosciences, Reproductive Sciences and Odontostomatology, University of Naples “Federico II”, 80131 Naples, Italy; 5 Department of Urology, University of Pisa, Lungarno Pacinotti 43, 56126 Pisa, Italy

**Keywords:** robot-assisted radical prostatectomy, nerve-sparing, cold dissection, urinary continence, erectile function, thermal injury, prostate cancer, functional recovery, IIEF-5, minimally invasive surgery

## Abstract

**Background**: Cold dissection (CD) during nerve-sparing robot-assisted radical prostatectomy (nsRARP) in patients with prostate cancer has been proposed to improve functional outcomes by reducing the thermal damage to neurovascular bundles (NVBs). This study compares the impact of CD versus thermal dissection (TD) on postoperative early continence and erectile function recovery. **Methods**: A prospective comparative analysis was conducted on patients undergoing nsRARP, comparing CD and TD techniques. Continence was assessed at 15, 30, and 90 days, while erectile function was evaluated at 30, 90, and 180 days using IIEF-5 scores. Logistic and linear regression analyses were performed to identify predictors of functional recovery. **Results**: CD significantly improved early continence at 15 and 30 days (*p* < 0.05), although the difference diminished by 90 days. Erectile function recovery was consistently higher in the CD group at all time points (*p* < 0.01). Age negatively impacted continence and erectile function recovery, while preoperative IIEF-5 scores were strong predictors of erectile function. Prostate volume, BMI, and metabolic syndrome showed no significant influence on outcomes. **Conclusions**: CD enhances early continence and erectile function recovery following nsRARP by preserving NVBs and minimizing thermal damage. This technique offers a valuable approach to improving patient functional outcomes. Further studies are required to validate its long-term benefits.

## Simple Summary

Robotic prostate surgery can cure prostate cancer, but many men experience urinary incontinence and erectile dysfunction afterward. This study investigated whether using a “cold dissection” technique during nerve-sparing robotic prostatectomy leads to better early recovery of urinary continence and sexual function. The researchers compared two groups of patients: one treated with cold dissection and the other with traditional thermal dissection. Patients treated with cold dissection had better urinary continence rates within the first 15 and 30 days and improved erectile function up to six months after surgery. This approach may help men recover faster and maintain a better quality of life after prostate cancer surgery.

## 1. Introduction

Prostate cancer (PCa) is the most prevalent solid tumor among men in Western countries. In the USA, of the 3.1 million newly diagnosed PCas between 2003 and 2017, 77% were localized cases [1]. In the next 20 years, it is estimated that there will be close to 2 million new cases [2]. Radical prostatectomy (RP) represents a key primary treatment option for managing PCa. Urinary incontinence (UI), more specifically stress urinary incontinence (SUI), and erectile dysfunction (ED) represent common long-term complications after RP and greatly impact patients’ quality of life (QoL) [3,4,5], regardless of surgical approach. Various factors have been reported to affect SUI and erectile function (EF) recovery following robot-assisted RP (RARP). These include patient-specific factors such as age, body mass index (BMI), diabetes mellitus, lower urinary tract symptoms (LUTSs), prostate volume, previous prostatic surgery, surgeon’s experience, and surgical technique [6,7,8]. Several studies have focused on surgical technique, representing the only modifiable factor in achieving better urinary continence (UC) [9,10,11,12,13,14,15], and EF [16,17,18,19,20]. The advent of RARP has granted improved and magnified optics, thus granting the possibility of more precise surgery by reducing blood loss and improving the visualization of the crucial anatomical structures that need preserving for better functional outcomes (FOs). The improved visualization consequently helps to preserve the neurovascular bundles (NVBs), thus resulting in improved nerve-sparing RARP (nsRARP), which represents a crucial step in improving FOs [21]. However, the lateral temperature spread of thermal devices, such as monopolar scissors and bipolar forceps, may cause damage to the NVBs (e.g., neurapraxia, axonotmesis, or neurotmesis). This iatrogenic injury can delay functional recovery despite successful oncologic resection during the dissection of the vascular pedicles (VPs), correlating with a longer functional recovery time [22,23]. The early restoration of UC and EF is critical for improving QoL, enabling a faster return to daily activities, and reducing psychosocial stress. In this context, we hypothesized that cold dissection would improve early UC and EF recovery compared to thermal dissection. Thus, our study aimed to investigate whether our cold dissection (CD) approach on the VPs during nsRARP for patients with localized PCa improves early UC and EF recovery, compared to touch thermal dissection (TD), also referred as touch cautery.

## 2. Materials and Methods

### 2.1. Study Design and Surgical Technique

From May 2021 to January 2024, we prospectively enrolled patients who underwent intrafascial nsRARP. This prospective comparative study was approved on 12 October 2020 by the Institutional Review Board and Ethics Committee of the Department of Medical and Surgical Sciences and Biotechnologies of Sapienza University in Rome (UROUNIVLT_OCT20/5821). The principles of the Declaration of Helsinki were followed, and written informed consent was obtained from all participants. The inclusion criteria included men under 70 years old with low-risk and intermediate-risk localized PCa (ISUP I, II), who were eligible for bilateral nsRARP, according to the European Association of Urology (EAU) guidelines [24], and refused active surveillance. Seventy years old was set as a cut-off to minimize age-related bias in functional recovery, as age is a negative predictor for functional recovery. The exclusion criteria included the following: men with high-risk PCa, previous prostatic surgery, pelvic radiation therapy, previous diagnosis of UI, and an International Index of Erectile Function (IIEF-5) score < 10 or previous therapy with Phosphodiesterase-5 Inhibitors (PDE5is). Various patient data were assessed, including age, BMI, medical [diabetes mellitus (DM), metabolic syndrome (MS)] and surgical history, home therapy, IIEF-5, hematochemical examinations [prostate-specific antigen (PSA), hemoglobin (Hb), and preoperative–postoperative Hb difference ∆Hb], ISUP grade at prostate biopsy, and prostatic volume at multiparametric magnetic resonance imaging (mpMRI). MS was defined as the presence of ≥3 of the following: waist circumference > 102 cm, triglycerides ≥ 150 mg/dL, HDL cholesterol < 40 mg/dL, blood pressure ≥ 130/85 mmHg or use of antihypertensive medication, and fasting glucose ≥ 110 mg/dL or diagnosis of DM [25].

**Figure 1:**
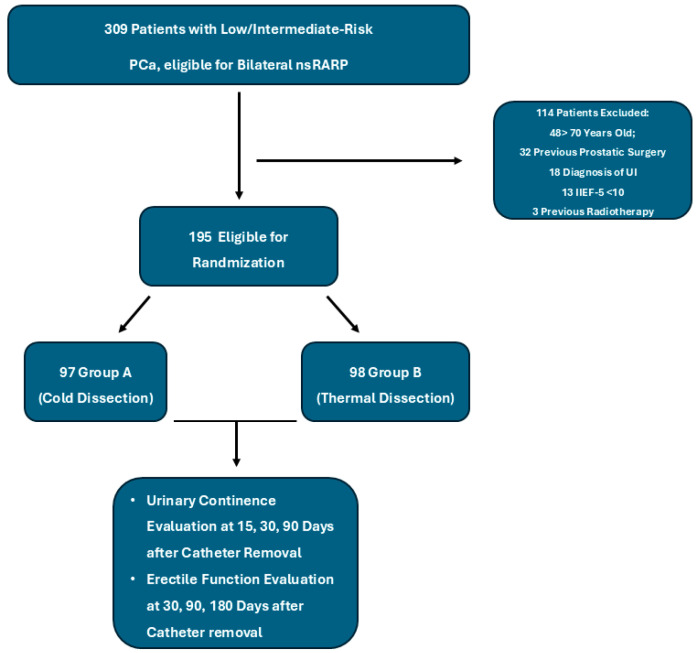
Flowchart illustrating patient selection, exclusion criteria, and randomization process. Of 309 patients with low- or intermediate-risk prostate cancer eligible for bilateral nerve-sparing robot-assisted radical prostatectomy (nsRARP), 114 were excluded due to age > 70 years, previous prostatic surgery, urinary incontinence, low baseline erectile function (IIEF-5 < 10), or prior radiotherapy. A total of 195 patients were randomized to receive either cold dissection (Group A, n = 97) or thermal dissection (Group B, n = 98). Continence was assessed at 15, 30, and 90 days, and erectile function at 30, 90, and 180 days post catheter removal. Abbreviations: nsRARP = nerve-sparing robot-assisted radical prostatectomy; IIEF-5 = 5-item International Index of Erectile Function; and PCa = prostate cancer.

The patients, randomized in a 1:1 ratio using SAS 9.4 software, were divided into two groups based on the dissection technique; no blinding was performed. CD was defined as vascular pedicle dissection using sharp scissors and hemostasis with hem-o-lok clips, without thermal energy. TD employed the brief activation of monopolar or bipolar energy sources (cut or coagulation modes) for vessel control. Dissections followed an intrafascial anatomical plane in both groups. The study was performed in accordance with the Ethical Principles for Medical Research Involving Human Subjects (World Medical Association, The Declaration of Helsinki Principles, 2000). One experienced surgeon (A.F.) employed the Montsouris [26] technique to perform RARP with the “da Vinci X” Surgical System. This technique involved incising the pouch of Douglas to dissect the vas deferens and seminal vesicles. VPs were then dissected with an intrafascial anatomical technique, utilizing small hem-o-lok clips and CD for group A and a combination of touch bipolar and monopolar cautery (TD) in group B to achieve hemostasis. After the completion of the posterior dissection, anterior dissection is performed by accessing the Retzius space, opening the endopelvic fascia, dissecting the bladder neck, dissecting the remaining VP with small hem-o-lok clips and CD in group A and with TD in group B, and dissecting the apex and the urethra. The vesicourethral anastomosis was performed using the Van Velthoven technique. All patients had their catheter removed between 4 and 7 days postoperatively. Continence was evaluated at 15, 30, and 90 days after catheter removal during regular outpatient visits. Postoperative UC was defined as the use of 0 pads/day. While no pad weight or validated questionnaires were used, this binary definition remains standard in contemporary urologic studies [27]. Furthermore, all patients received PDE5is at catheter removal, and EF was evaluated with IIEF-5 at 30, 90, and 180 days; patients were considered responders if IIEF-5 scores reached or exceeded 17. UC typically stabilizes within 3 months, while EF may recover gradually over 6–24 months, necessitating a longer evaluation period. Figure 1 illustrates the study design.

### 2.2. Statistical Analysis

Statistical analyses were conducted using SPSS version 26.0 (IBM Corp., Armonk, NY, USA). Continuous variables were expressed as means and standard deviations (SDs) and compared between groups using Student’s *t*-test. Categorical variables were reported as percentages and analyzed using the chi-square test or Fisher’s exact test. Logistic regression analysis was performed to identify predictors of continence recovery at 15, 30, and 90 days postoperatively. The results were reported as odds ratios (ORs) with 95% confidence intervals (CIs), and significance was defined as *p* < 0.05. For erectile function recovery, linear regression models were used to evaluate the predictors of postoperative IIEF-5 scores at 30, 90, and 180 days. Regression coefficients (β) and their corresponding 95% CIs were reported. All tests were two-tailed, and a *p*-value < 0.05 was considered statistically significant. While an a priori sample size calculation was not conducted, a post hoc power analysis demonstrated that the study had over 80% power to detect a 15% difference in continence rates at the 30-day follow-up.

## 3. Results

### 3.1. Preoperative Characteristics

The study included 195 patients, 97 of whom underwent RARP with the CD technique (Group A), and 98 of whom underwent RARP with the TD technique (Group B). The patients’ preoperative characteristics were comparable between the groups, as shown in Table 1. Patients in Group A had a higher mean age than in Group B (63.26 ± 5.69 vs. 61.14 ± 4.51 years, *p* = 0.07). No statistically significant differences were observed between the groups in terms of BMI (Group A 22.67 ± 2.46 vs. Group B 22.69 ± 2.74, *p* = 0.42), prevalence of DM (17.53% vs. 19.39%, *p* = 0.84), or MS (Group A 21.65% vs. Group B 24.49%, *p* = 0.76). Prostate volume was comparable between the groups (Group A 48.11 ± 10.4 mL vs. Group B 50.09 ± 10.98 mL, *p* = 0.28), as were the PSA levels (Group A 6.4 ± 1.55 ng/mL vs. Group B 6.65 ± 1.98 ng/mL, *p* = 0.457). The distribution of ISUP grades at biopsy did not differ significantly, with ISUP grade 1 observed in 46.39% of patients in Group A versus 55.1% in Group B (*p* = 0.28), and ISUP grade 2 observed in 53.61% and 44.9% of patients, respectively (*p* = 0.28). The Hb levels were significantly higher in Group B compared to Group A (14.5 ± 1.38 g/dL vs. 14.1 ± 0.81 g/dL, *p* = 0.041). The preoperative IIEF-5 scores were comparable between the groups (Group A 24.68 ± 2.75 vs. Group B 24.45 ± 2.64, *p* = 0.54).

### 3.2. Postoperative Outcomes

**Figure 2:**
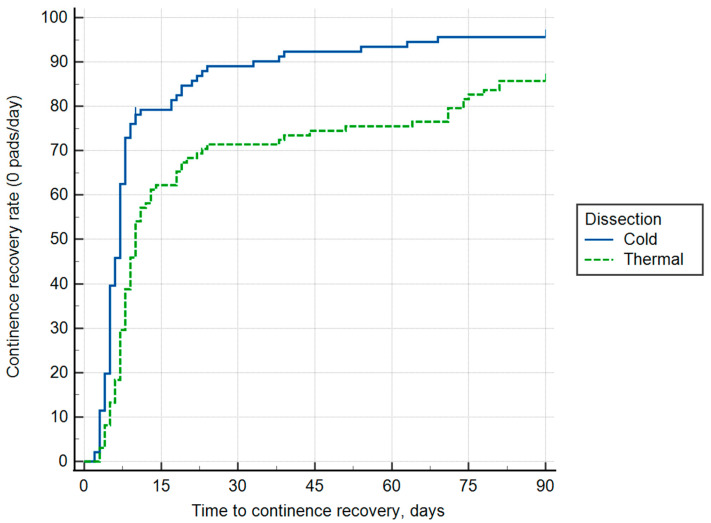
Kaplan–Meier curve showing time to continence recovery (defined as 0 pads/day) following radical prostatectomy, stratified by dissection technique. Patients in the cold dissection group (solid blue line) exhibited significantly faster recovery at 15 and 30 days after catheter removal (log-rank *p* < 0.05) than those in the thermal dissection group (dashed green line), while continence rates converged between groups by 90 days.

The operative time was similar between Group A and Group B, with a mean of 142.4 ± 16 and 138.1 ± 15.54 min in Groups A and B, respectively. Furthermore, the ΔHb was less in Group B (1.53 ± 0.7 g/dL vs. 1.61 ± 0.8 g/dL, *p* = 0.08), but no statistically significant difference was found. The positive surgical margin (PSM) rate was comparable between the groups (Group A 9.59% vs. Group B 9.18%, *p* = 0.28). The time to catheter removal was not significantly different, with Group A showing a mean time of 5.3 ± 0.7 days compared to 5.9 ± 0.9 days in Group B (*p* = 0.31). The distribution of ISUP grades was balanced, with ISUP grade 1 observed in 44.33% and 52.04% in groups A and B, respectively, and ISUP grade 2 in 51.55% vs. 44.89% in groups A and B, respectively. Tumor upstaging was evident in 4.12% and 3.07% (ISUP grade 3) in groups A and B, respectively (*p* = 0.39). Pathological staging was not significantly different between the groups, with T2a accounting for 11.34% vs. 13.27% (*p* = 0.42), T2b for 38.14% vs. 40.82% (*p* = 0.56), T2c for 45.36% vs. 41.84% (*p* = 0.83), and T3a for 5.15% vs. 4.08% (*p* = 0.75), in groups A and B, respectively. Continence recovery was significantly faster in Group A (Figure 2).

At 15 days after catheter removal, 78.35% of patients in Group A were continent compared to 62.24% in Group B (*p* = 0.014). By 30 days, continence rates were 87.62% in Group A vs. 71.43% in Group B (*p* = 0.023), and at 90 days, continence rates were higher in Group A (93.81% vs. 85.71%), but this difference was not statistically significant (*p* = 0.17). The IIEF-5 scores were better in Group A at all postoperative time points. At 30 days, Group A had a mean IIEF-5 score of 7.68 ± 2.45 compared to 5.66 ± 2.59 in Group B (*p* = 0.0061). At 90 days, scores were 10.13 ± 2.47 in Group A vs. 7.38 ± 2.49 in Group B (*p* = 0.0057). By 180 days, the EF scores had improved further, with Group A achieving a mean of 15.55 ± 2.94 compared to 12.73 ± 2.95 in Group B (*p* = 0.0069). Table 2 summarizes the postoperative outcomes.

### 3.3. Regression Analysis for Continence Recovery

Logistic regression analysis was conducted to identify predictors of continence recovery at 15, 30, and 90 days postoperatively, the results of which are summarized in Table 3. Dissection technique and age were statistically significant predictors of continence recovery. In particular, TD was associated with significantly lower odds of continence recovery compared to CD at the 15-day follow-up (OR = 0.49, 95% CI: 0.25–0.97, *p* = 0.04) and the 30-day follow-up (OR = 0.4, 95% CI: 0.18–0.89, *p* = 0.02), while at the 90-day follow-up, it was not found to be a statistically significant predictor. Age was a significant predictor at the 15-day (OR = 0.92, 95% CI: 0.86–0.99, *p* = 0.03), 30-day (OR = 0.92, 95% CI: 0.85–0.99, *p* = 0.04), and 90-day follow-up (OR = 0.84, 95% CI: 0.75–0.95, *p* = 0.007). The other variables (BMI, prostate volume, and MS) were not clinically significant.

### 3.4. Linear Regression Analysis for Erectile Function Recovery

Linear regression was used to identify predictors of postoperative EF recovery, as assessed using IIEF-5 scores at 30, 90, and 180 days. The results are summarized in Table 4. Dissection technique, preoperative IIEF-5 scores, and age were statistically significant predictors of erectile function recovery. In particular, CD was associated with significantly higher IIEF-5 scores compared to TD at the 30-day follow-up (β = 1.86, 95% CI: 1.11–2.60, *p* = 0.0094), 90-day follow-up (β = 2.57, 95% CI: 1.86–3.28, *p* = 0.0055), and 180-day follow-up (β = 2.7, 95% CI: 1.83–3.55, *p* = 0.0092). Preoperative IIEF-5 scores were also significant predictors across all time points, with higher preoperative scores strongly associated with improved erectile function recovery at 30 days (β = 0.38, 95% CI: 0.26–0.50, *p* = 0.0075), 90 days (β = 0.41, 95% CI: 0.29–0.52, *p* = 0.0041), and 180 days (β = 0.43, 95% CI: 0.30–0.56, *p* = 0.0066). Age was found to be a significant negative predictor of erectile function recovery at the 30-day (β = −0.09, 95% CI: −0.16 to −0.02, *p* = 0.02) and 90-day follow-ups (β = −0.09, 95% CI: −0.16 to −0.01, *p* = 0.01), but not at the 180-day follow-up. The other variables considered, including prostate volume, BMI, and MS, were not found to be statistically significant predictors at any time point.

### 3.5. Erectile Function Responder Analysis

A multivariable logistic regression analysis was conducted to identify independent predictors of patients who recovered EF at 180 days (IIEF-5 ≥ 17). CF remained significantly associated with higher odds of recovery (OR: 0.04; 95% CI: 0.00–0.36; *p* = 0.006), and baseline IIEF-5 also represented a strong predictor (OR: 1.63; 95% CI: 1.18–2.26; *p* = 0.003). MS, age, BMI, DM, and prostate volume were not statistically significant predictors (all *p* > 0.1). These findings confirm the dominant role of the surgical technique and preoperative functional status in early erectile recovery. Table 5 summarizes the regression results.

## 4. Discussion

Prostate cancer represents the most common solid neoplasm in the male population [2,28], and RP represents a valid therapeutic option for low-, intermediate-, and high-risk localized PCa in patients with a life expectancy of >10 years [24]. The development of RARP has completely changed the landscape of prostatic surgery, with over 80% of procedures being RARP by 2012 [29]. Despite the technical advancements, such as stable magnified 3D images, tremor canceling instruments with a 7° of freedom EndoWrist, and motion scaling [30], RARP and RP, in general, are associated with UI and ED that may significantly impair the patient’s QoL [31], with UI rates ranging from 4% to 50% [27,32], and ED rates of up to 70% [33]. Furthermore, due to the popularity of robot-assisted surgery, patient expectations have shifted from oncological control to functional recovery [34]. A consensus among urologists is that NVB preservation is paramount for functional recovery [21,35]. Anatomic studies have shown that periprostatic nerves are dispersed over the ventrolateral and dorsal surfaces of the prostate, extending anteriorly on the lateral prostate [36,37]. Nerve injuries were first classified by Seddon [38] into the following categories:

**Neurapraxia**: a first-degree injury that may occur following mechanical trauma; the expected recovery time is approximately 12 months;

**Axonotmesis**: a second-degree injury that affects the axon while preserving the surrounding connective tissue; functional recovery is expected within 24 months;

**Neurotmesis**: The most severe type of injury, resulting in permanent functional loss.

Furthermore, nerve damage also depends on the nature of the nerve structures. Some studies described the presence of both myelinated and unmyelinated structures in this anatomical region [39]. The nerves within the NVB appear to be sensitive to thermal energy spreading from nearby structures during energy use [40]. In particular, the most susceptible portions to thermal injury seem to be located at the prostatic apex. In this region, the nerves are located around the urethra, in an area where maintaining enough distance to prevent thermal spread is challenging [41]. A study by Dagtekin et al. on animal models tested and quantified the effects of electrocauterization through electroneurography. The study revealed a sharp decline in nerve function in the weeks following treatment. This effect was most pronounced during the first week, followed by a slow recovery over three weeks [42]. Over the years, surgical techniques that avoid monopolar and bipolar energy have progressively emerged, leading to the development of cautery-free procedures capable of achieving adequate hemostasis, such as the use of vascular clips, surgical ligatures, and suturing clamps [43]. Studies investigating the type of energy used in surgery indicate that both monopolar and bipolar energy damage nervous structures. However, the risk of nerve damage seems to decrease when performing the touch electrocautery technique with bipolar energy compared to monopolar energy [44]. In this study, we aimed to evaluate if our energy-free approach to the VPs may lead to faster functional recovery compared to touch electrocautery. The results indicate that energy-free CD significantly improves both early UC and EF recovery compared to TD. These findings align with prior studies that emphasize the importance of preserving the NVBs and reducing surgical trauma to periprostatic tissues. At the 15- and 30-day follow-ups, CD was associated with significantly higher odds of UC recovery. This effect may be attributed to the reduced lateral thermal spread with the CD technique, minimizing damage to the structures critical for continence. By 90 days, the difference in continence rates between the two groups was no longer statistically significant. This suggests that while CD facilitates early recovery, patient-specific characteristics and long-term healing may equalize outcomes over a longer time period. A recent retrospective study by Hofman et al. [20] found that touch electrocautery was not inferior to athermal dissection, even though the same group demonstrated that electrical transmission induced thermal damage in animal models. This damage elevated the surrounding parenchyma temperature to above 43–45 °C, exacerbated in areas with poor blood supply. In such regions, temperatures can rapidly rise by 10–14 °C without effective heat dissipation [44,45]. Additionally, a recent prospective randomized study by Taille suggested that using low-intensity, short-duration bipolar energy produced functional outcomes similar to those achieved with athermal dissection techniques, suggesting that the duration of tissue exposure to energy is a critical factor in triggering nerve damage in adjacent structures [46]. In our study, we observed no significant statistical differences in urinary continence recovery between TD and CD at the 90-day follow-up, suggesting that careful energy use does not affect long-term continence recovery. Age emerged as a negative predictor of UC recovery, reflecting the impact of the age-related decline in pelvic floor muscle strength and healing capacity. EF recovery also showed a clear benefit for the CD group at all postoperative time points. The significantly higher IIEF-5 scores in the CD group at 30, 90, and 180 days underscore the protective effect of this technique on NVBs. CD likely reduces the thermal injury and mechanical stress on these delicate structures, allowing for the faster and more robust recovery of EF. Preoperative IIEF-5 scores represented a significant predictor, highlighting the importance of the baseline EF in determining postoperative outcomes. Age was a negative predictor at 30 and 90 days, but had a diminished effect by 180 days, suggesting that while older patients recover more slowly, they can still achieve functional improvement over time. In our responder analysis, CD and a higher baseline IIEF-5 score were the strongest predictors of EF recovery at 180 days. CD was associated with markedly improved odds of recovery, underscoring its potential neuroprotective benefit. The high positive surgical margin rate represents one drawback of athermal approaches [47], confirmed by our study, even though this finding was not statistically significant and was comparable between both groups. In conclusion, while CD seems to offer advantages in the early postoperative period, these seem to be mitigated in the long term. Further studies are needed to investigate its long-term effects and validate these findings across larger, more diverse patient populations. The limitations of this study include its single-center design, relatively short follow-up, and lack of patient-reported satisfaction outcome measures. Prospective, multicenter trials with extended follow-up are necessary to confirm the durability of these benefits and assess the potential differences in oncological outcomes. This study was performed by a single high-volume surgeon, which standardizes the technique, but may limit generalizability to broader surgical practice. Furthermore, future research should explore patient-reported outcomes and QoL measures to provide a comprehensive understanding of the impact of CD on overall recovery following RARP.

## 5. Conclusions

In our study, cold dissection appears to enhance early postoperative functional recovery in patients undergoing nsRARP. These findings suggest that minimizing thermal injury may enhance early functional outcomes without compromising oncologic safety. However, the benefits observed at 90 and 180 days warrant further validation in studies with extended follow-up.

Prospective, multicenter studies, with a more diverse population and with extended follow-up, are essential to evaluate the durability of these functional improvements and to explore potential differences in oncological outcomes. Additionally, incorporating patient-reported outcome measures and QoL assessments will be crucial to understand the broader impact of this technique on postoperative recovery. The relatively short observation period may limit our ability to fully capture long-term functional recovery and QoL trajectories. Future longitudinal studies should be planned to assess whether these early advantages are sustained beyond one year, to better inform surgical decision-making and guide patient counseling.

## Figures and Tables

**Table 1:** Baseline demographic and clinical characteristics of patients undergoing nerve-sparing robot-assisted radical prostatectomy (nsRARP) with cold dissection (Group A, n = 97) or thermal dissection (Group B, n = 98). Values are reported as mean ± standard deviation (SD) or percentages. No significant differences were observed between groups at baseline besides preoperative Hb levels. Abbreviations: BMI = body mass index; DM = diabetes mellitus; MS = metabolic syndrome; PSA = prostate-specific antigen; ISUP = International Society of Urological Pathology; Hb = hemoglobin; IIEF-5 = 5-item International Index of Erectile Function; and SD = standard deviation.

Variables	Group A (97 Patients)	Group B (98 Patients)	*p*
Age, yy (SD)	63.26 (5.69)	61.14 (4.51)	0.07
BMI, kg/m^2^ (SD)	22.67 (2.46)	22.69 (2.74)	0.42
DM, %	17.53	19.39	0.84
MS, %	21.65	24.49	0.76
Prostate Volume, mL (SD)	48.11 (10.4)	50.09 (10.98)	0.28
PSA, ng/mL (SD)	6.4 (1.55)	6.65 (1.98)	0.457
**Grade Group:**			
ISUP 1, %	46.39	55.1	0.28
ISUP 2, %	53.61	44.9	0.28
Preoperative Hb, g/dL (SD)	14.1 (0.81)	14.5 (1.38)	0.041
IIEF-5, mean (SD)	24.68 (2.75)	24.45 (2.64)	0.54

**Table 2:** Comparison of perioperative parameters and early functional outcomes between patients treated with cold versus thermal dissection. Significant differences favoring cold dissection were observed in early continence and erectile function scores. Abbreviations: Hb = hemoglobin; ΔHb = hemoglobin difference (pre- vs. postoperative); IIEF-5 = 5-item International Index of Erectile Function; ISUP = International Society of Urological Pathology; and SD = standard deviation. Bold indicates *p*-values and grouping of variables.

Variables	Group A (97 Patients)	Group B (98 Patients)	*p*
Operative Time, min (SD)	142.4 (16)	138.1 (15.54)	**0.1**
Estimated Blood Loss, mL (SD)	267.37 (47.04)	261.51 (46.38)	**0.38**
Postoperative Hb, g/dL (SD)	12.49 (0.85)	13.02 (1.34)	**0.07**
ΔHb, g/dL (SD)	1.61 (0.8)	1.53 (0.7)	**0.08**
Positive Surgical Margin, %	9.59	9.18	**0.28**
Catheter Removal Time, days (SD)	5.3 (0.7)	5.9 (0.9)	**0.31**
**Grade Group**			
ISUP 1, %	44.33	52.04	**0.31**
ISUP 2, %	51.55	44.89	**0.27**
ISUP 3, %	4.12	3.07	**0.39**
**Pathological Tumor Stage**			
T2a, %	11.34	13.27	**0.42**
T2b, %	38.14	40.82	**0.56**
T2c, %	45.36	41.84	**0.83**
T3a, %	5.15	4.08	**0.75**
**Postoperative Continence**			
15 Days, %	78.35	62.24	**0.014**
30 Days, %	87.62	71.43	**0.023**
90 Days, %	93.81	85.71	**0.17**
**Postoperative Erectile Function**			
IIEF-5 30 Days (SD)	7.68 (2.45)	5.66 (2.59)	**0.** **0061**
IIEF-5 90 Days (SD)	10.13 (2.47)	7.38 (2.49)	**0.** **0057**
IIEF-5 180 Days (SD)	15.55 (2.94)	12.73 (2.95)	**0.** **0069**

**Table 3:** Multivariable logistic regression models identifying predictors of urinary continence recovery at 15, 30, and 90 days after catheter removal. Abbreviations: β = regression coefficient; OR = odds ratio; CI = confidence interval; BMI = body mass index; MS = metabolic syndrome; DM = diabetes mellitus; and PSA = prostate-specific antigen. Bold indicates *p*-values.

Continence at 15-day Follow-up					
**Predictor**	**β**	***p***	**OR**	**CI Lower 95%**	**CI Upper 95%**
Dissection	−0.7	**0.04**	0.49	0.25	0.97
Age	−0.07	**0.03**	0.92	0.86	0.99
Prostate Volume	0.02	**0.099**	1.02	0.99	1.05
BMI	−0.09	**0.15**	0.9	0.8	1.03
MS	−0.22	**0.65**	0.79	0.29	2.18
**Continence at 30-day Follow-up**					
**Predictor**	**β**	***p***	**OR**	**CI Lower 95%**	**CI Upper 95%**
Dissection	−0.9	**0.02**	0.4	0.18	0.89
Age	−0.08	**0.04**	0.92	0.85	0.99
Prostate Volume	0.03	**0.07**	1.03	0.99	1.06
BMI	−0.037	**0.61**	0.96	0.83	1.11
MS	−0.01	**0.97**	0.98	0.3	3.2
**Continence at 90-day Follow-up**					
**Predictor**	**β**	***p***	**OR**	**CI Lower 95%**	**CI Upper 95%**
Dissection	−0.7	**0.2**	0.48	0.16	1.46
Age	−0.16	**0.007**	0.84	0.75	0.95
Prostate Volume	0.04	**0.13**	1.04	0.98	1.09
BMI	0.03	**0.77**	1.02	0.83	1.26
MS	−0.17	**0.83**	0.84	0.15	4.48

**Table 4:** Multivariable linear regression models identifying predictors of erectile function recovery (measured based on IIEF-5 scores) at 30, 90, and 180 days after catheter removal. Abbreviations: β = regression coefficient; CI = confidence interval; IIEF-5 = 5-item International Index of Erectile Function; BMI = body mass index; MS = metabolic syndrome; Hb = hemoglobin; and PSA = prostate-specific antigen. Bold indicates *p*-values.

Erectile Function at 30-day Follow-up				
**Predictor**	**β**	***p***	**CI Lower 95%**	**CI Upper 95%**
Dissection	1.86	**0.** **0094**	1.11	2.6
Age	−0.09	**0.02**	−0.16	−0.02
Prostate Volume	0.01	**0.33**	−0.01	0.04
BMI	0.01	**0.86**	−0.13	0.16
MS	−0.21	**0.71**	−1.36	0.94
Preoperative IIEF-5	0.38	**0.** **0075**	0.26	0.5
**Erectile Function at 90-day Follow-up**				
**Predictor**	**β**	***p***	**CI Lower 95%**	**CI Upper 95%**
Dissection	2.57	**0.** **0055**	1.86	3.28
Age	−0.09	**0.01**	−0.16	−0.01
Prostate Volume	0.02	**0.13**	−0.01	0.05
BMI	0.03	**0.61**	−0.1	0.17
MS	0.43	**0.44**	−0.67	1.53
Preoperative IIEF-5	0.41	**0.** **0041**	0.29	0.52
**Erectile Function at 180-day Follow-up**				
**Predictor**	**β**	***p***	**CI Lower 95%**	**CI Upper 95%**
Dissection	2.7	**0.** **0092**	1.83	3.55
Age	−0.08	**0.08**	−0.16	0.009
Prostate Volume	0.025	**0.14**	−0.08	0.06
BMI	0.04	**0.65**	−0.13	0.21
MS	0.65	**0.33**	−0.7	1.9
Preoperative IIEF-5	0.43	**0.** **0066**	0.30	0.56

**Table 5:** Multivariable logistic regression analysis of factors associated with erectile function recovery, defined as achieving IIEF-5 ≥ 17 at 180 days after catheter removal. Abbreviations: β = regression coefficient; OR = odds ratio; CI = confidence interval; IIEF-5 = 5-item International Index of Erectile Function; BMI = body mass index; MS = metabolic syndrome; and DM = diabetes mellitus.

Responder Analysis					
**Predictor**	**β**	***p***	**OR**	**CI Lower 95%**	**CI Upper 95%**
Dissection	−3.17	**0.006**	0.04	0.001	0.4
Age	0.09	**0.234**	1.1	0.94	1.28
BMI	0.048	**0.725**	1.05	0.8	1.37
DM	−0.89	**0.418**	0.41	0.05	3.55
MS	0.458	**0.597**	1.58	0.29	8.64
Prostate Volume	−0.003	**0.894**	1.0	0.95	1.04
Preoperative IIEF-5	0.48	**0.003**	1.63	1.18	2.26

## Data Availability

The data are unavailable due to privacy or ethical restrictions imposed by our institution.

## Abbreviations

BMI	Body mass index
CD	Cold dissection
CI	Confidence interval
ΔHb	Change in hemoglobin (preoperative–postoperative)
DM	Diabetes mellitus
ED	Erectile dysfunction
EF	Erectile function
FOs	Functional outcomes
Hb	Hemoglobin
IIEF-5	International Index of Erectile Function, 5-item version
ISUP	International Society of Urological Pathology
LUTSs	Lower urinary tract symptoms
MPMRI	Multiparametric magnetic resonance imaging
MS	Metabolic syndrome
NSRARP	Nerve-sparing robot-assisted radical prostatectomy
NVB	Neurovascular bundle
OR	Odds ratio
PCa	Prostate cancer
PDE5i	Phosphodiesterase-5 Inhibitors
PSA	Prostate-specific antigen
PSM	Positive surgical margin
QoL	Quality of life
RARP	Robot-assisted radical prostatectomy
RP	Radical prostatectomy
SD	Standard deviation
SUI	Stress urinary incontinence
TD	Thermal dissection
UI	Urinary incontinence
UC	Urinary continence
